# Integrating Sex/Gender into Environmental Health Research: Development of a Conceptual Framework

**DOI:** 10.3390/ijerph182212118

**Published:** 2021-11-18

**Authors:** Gabriele Bolte, Katharina Jacke, Katrin Groth, Ute Kraus, Lisa Dandolo, Lotta Fiedel, Malgorzata Debiak, Marike Kolossa-Gehring, Alexandra Schneider, Kerstin Palm

**Affiliations:** 1 Department of Social Epidemiology, Institute of Public Health and Nursing Research, University of Bremen, 28359 Bremen, Germany; dandolo@uni-bremen.de; 2 Health Sciences Bremen, University of Bremen, 28359 Bremen, Germany; 3 Gender and Science Research Unit, Institute of History, Humboldt-University of Berlin, 10099 Berlin, Germany; katharina.jacke@hu-berlin.de (K.J.); lotta-lili.fiedel@uni-oldenburg.de (L.F.); kerstin.palm@hu-berlin.de (K.P.); 4 Section II 1.2 Toxicology, Health-Related Environmental Monitoring, German Environment Agency, 14195 Berlin, Germany; Katrin.Groth@senbjf.berlin.de (K.G.); malgorzata.debiak@uba.de (M.D.); marike.kolossa@uba.de (M.K.-G.); 5 Institute of Epidemiology, Helmholtz Zentrum München, German Research Center for Environmental Health (GmbH), 85764 Neuherberg, Germany; ute.kraus@helmholtz-muenchen.de (U.K.); alexandra.schneider@helmholtz-muenchen.de (A.S.)

**Keywords:** gender, sex, intersectionality, embodiment, health equity, inequality, social determinants, environment, concept, model, framework

## Abstract

There is a growing awareness about the need to comprehensively integrate sex and gender into health research in order to enhance the validity and significance of research results. An in-depth consideration of differential exposures and vulnerability is lacking, especially within environmental risk assessment. Thus, the interdisciplinary team of the collaborative research project INGER (integrating gender into environmental health research) aimed to develop a multidimensional sex/gender concept as a theoretically grounded starting point for the operationalization of sex and gender in quantitative (environmental) health research. The iterative development process was based on gender theoretical and health science approaches and was inspired by previously published concepts or models of sex- and gender-related dimensions. The INGER sex/gender concept fulfills the four theoretically established prerequisites for comprehensively investigating sex and gender aspects in population health research: multidimensionality, variety, embodiment, and intersectionality. The theoretical foundation of INGER’s multidimensional sex/gender concept will be laid out, as well as recent sex/gender conceptualization developments in health sciences. In conclusion, by building upon the latest state of research of several disciplines, the conceptual framework will significantly contribute to integrating gender theoretical concepts into (environmental) health research, improving the validity of research and, thus, supporting the promotion of health equity in the long term.

## 1. Introduction

There is a growing awareness of the need to integrate sex and gender more comprehensively into health research to enhance the validity and significance of research results providing the evidence basis for prevention measures, health promotion, and health care [1,2,3,4,5]. Nevertheless, until now, sex and gender “as a domain of complex phenomena that are simultaneously biological and social” [6] (p. 1818) has, to a large extent, not been part of environmental health research [7,8,9,10,11]. According to previous reviews, this especially applies to the disciplines environmental epidemiology [12] and environmental toxicology [13,14,15], and, to a lesser extent, to environmental public health research [7,9].

So far, the environmental health paradigm is predominantly based upon the distinction of sex-linked biological characteristics (organs and physiology of the reproductive system [16]). The dichotomous category “male/female” is used for stratification or even merely for adjusting as confounder in multivariable analyses, assuming static differences between men and women on an individual level. In sex-disaggregated analyses, the variable “sex” is often misleadingly interpreted as a proxy for gender. Or vice versa, the term “gender” is used to describe sex-linked biological phenomena [8].

However, against the background of current gender theoretical research, the term gender refers to social and structural factors [6], for example power relations in a society, living conditions, and socially influenced behaviors. It is also, therefore, linked to exposure conditions, such as activity patterns, places of stay, eating habits, or use of cosmetics and consumables. Moreover, gender-related factors and stressors (e.g., experience of discrimination, social role relations, norms) may modify the health effects of environmental exposures [7,12,13,14].

Concerning environmental risk assessment, Schwartz et al. [17] emphasize the need to pay greater attention to the clustering of environmental risks as well as differential vulnerability, both of which are assumed to be the consequences of social, political, and/or economic processes. Although the authors do not explicitly mention gender in a second article on the potential sources of this differential vulnerability and susceptibility to the health effects of environmental exposures [18], the highlighted aspects of socioeconomic position, psychosocial stressors, and allostatic load are known to be connected to gender-related living conditions. Cantarero and Aguirre [10] summarize the hitherto available evidence on sex or gender inequities in environment and health, and conclude that sex or gender differences in environmental conditions, exposures, and the health effects of these occur, as well as possible interactions of sex and gender with other social stratifications relevant for differential susceptibility. Thus, if sex and gender aspects are not adequately studied, both in exposure assessment and in analyses of health effects, the evidential basis needed to develop sex and gender-specific measures for environmental health protection and health promotion will remain inadequate.

Research from various disciplines (biology, medicine, public health, gender studies) emphasizes various prerequisites needed to comprehensively study sex and gender aspects in population health research. These can be subdivided into four prerequisites under the following terms: multidimensionality, variety, embodiment, and intersectionality.

(1) **Multidimensionality**. Sex and gender categories have been developed as concepts with different dimensions. The gender category, in particular, includes power relations, as put forward by Sandra Harding. She distinguishes between three levels of the gender system as an analytical basis for the different dimensions. The levels have since become established in gender studies: the individual, the structural, and the symbolic [19]. She refers to them as “a pivotal way in which humans identify themselves as persons (individual gender or gender identity), organize social relations (gender structure) and symbolize meaningful natural and social events and processes (gender symbolism)” [19] (p. 18).

Sex, and more specifically the term “sex-linked biological characteristics”, refers both to various “biological characteristics enabling sexual reproduction”, such as hormone levels and sexual organs [2] (p. 653), as to the embodied biological expression of gender (see embodiment), conceived by Nancy Krieger as the “gendered expressions of biology” [2] p. 653). Here, health research that is pragmatically tailored to its respective scientific concerns needs to clearly explain, in each case, which of these dimensions are being addressed [6,20].

(2) **Variety**. Sex/gender is not accurately captured by a static and dichotomous understanding of male/female [3,5,21]. There is an inherent variability within “sex” as a biological construct, encompassing genes, hormones, physiology, organs, and anatomy [21,22]. “Although conceptualizing sex usually relies on the female/male binary, in reality, individuals’ sex characteristics exist on a fluid and medically or socially constructed continuum. (…) Therefore, our common binary understanding of sex (male/female) is limiting and unrepresentative of the breadth and variety that exist with respect to human sex characteristics.” [22] (p. 3). The same holds for ‘gender’ as a “social construct that is culturally based and historically specific, and thus constantly changing. (…) The experience of gender is always linked to the social and political context.” [22] (p. 3).

(3) **Embodiment**. Somatic and social factors influence each other in a dynamic way and, therefore, cannot be conceptualized as independent of each other [2,23,24,25,26]. For example, gender socialization can affect bone and muscle growth through exercise and nutrition, which in turn may influence social positioning [27]. Ongoing experiences of discrimination may lead to psychosocial stress, with behavioral mechanisms, such as adverse health behaviors, and biological mechanisms in terms of allostatic load translating stress into disease [28,29]. Nancy Krieger defines embodiment as a multilevel phenomenon and active and reciprocal process regarding “how we literally incorporate, biologically, the material and social world in which we live” [25] (p. 352). Thus, when analyzing the health effects of exposures, attention must be given to the relevance of sex-linked biology and gender relations and their potential interactions [2].

We use the term “sex/gender” hereafter, as recommended by Springer et al. [6] to indicate the “irreducibly entangled phenomenon of ‘sex/gender’ “ [6] (p. 1818) as it is conceptualized by the embodiment theory. This allows us to avoid the conceptual muddle created by the interchangeable use of the concepts “sex” and “gender” in a dualistic and individualized way, apart from theory, as has been described for “gender-specific medicine” [30]. The term “sex/gender” does not imply the interchangeability of sex and gender, but indicates the necessity to examine specific mechanisms of the entanglement of sex and gender. Furthermore, we use the term “sex/gender-related” to emphasize the need to consider the multiple ways in which the relation between sex, gender, and health is shaped.

(4) **Intersectionality**. Intersectionality as a theoretical framework addresses the power related heterogeneity within the sex/gender categories (based on, for example, socioeconomic position, race/ethnicity, and/or sexual orientation) or within other social categories and considers inter- and intra-categorical complexity. An intersectionality-informed analysis contributes to the understanding of causes of health inequities and inequalities at individual and contextual levels, including multiple power relations and processes of discrimination [3,31,32,33]. Many definitions of intersectionality have in common that they emphasize that intersections of multiple social categories at the micro level “reflect multiple interlocking systems of privilege and oppression at the macro, social-structural level” [34] (p. 1267).

In alignment with these four prerequisites for comprehensively studying sex/gender aspects in population health research, the collaborative research project INGER (integrating gender into environmental health research) (https://www.uni-bremen.de/en/inger, accessed on 21 October 2021), aimed to develop a multidimensional sex/gender concept for quantitative environmental health research. The following paragraphs describe the iterative development process and the concept itself. The concept not only aims to guide operationalization for sex/gender-related data collection and analyses in population-based studies on environmental health within the framework of INGER, but may prove useful in other areas of population health research.

## 2. Materials and Methods

### 2.1. Collaborative Interdisciplinary Research Group

Within the INGER research group, expertise on environmental public health, environmental epidemiology, environmental toxicology, human biomonitoring, and gender studies was brought together to reach our goal through interdisciplinary research. INGER’s scientific advisory board consists of experts in gender and public health, gender medicine, gender and social-ecological research, environmental health research, science and technology studies, and feminist technoscience studies. INGER’s project team was able to draw upon the experiences and results of the preceding “research network sex/gender-environment-health (GeUmGe-NET)” [8].

### 2.2. Development of a Multidimensional Sex/Gender Concept from the Perspective of Intersectionality

A three-stage procedure was applied to develop a multidimensional sex/gender concept as a basis for a more comprehensive consideration of sex/gender in data collection and analysis.

#### 2.2.1. Stage One: An Inventory of Sex/Gender Concepts in Environmental Health Research

In the first stage, we assessed the current state of sex/gender conceptualization and operationalization in quantitative environmental health research. Previous reviews and conceptual publications on sex/gender and environmental health (research), e.g., [10,12], had already been identified by the preceding project GeUmGe-NET [8]. Within INGER, in systematic reviews, we analyzed whether sex/gender was considered at all and how sex/gender was conceptualized in prespecified selected topics on the impact of ambient air pollution on cardiovascular mortality [35], of environmental noise exposure on cardiovascular health [36], and of residential green space on self-rated health [9]. In addition, the questionnaires of five human biomonitoring studies were analyzed [37].

#### 2.2.2. Stage Two: An Inventory of Concepts or Models of Sex- and Gender-Related Dimensions

The evidence available so far from systematic reviews in the field of environmental health research, as mentioned in the previous section on the conceptualization of sex/gender and the operationalization of sex/gender concepts, showed no consideration of current gender theoretical concepts. Only the binary category male/female was used without a theoretical basis. Therefore, in the second stage, we collected literature comprising multidimensional concepts, models, instruments, indices, and tools related to the analytical categories of sex and gender across disciplines, which would lead to a more elaborated operationalization in quantitative health research. The method of our explorative approach of review and synthesis of theories was inspired by the work of Whitehead et al. [38].

Step 1: Database search. To find literature containing sound theory-based concepts of sex/gender for quantitative research, searches with a health science reference were conducted in PubMed (biomedical sciences) and PsycInfo (psychology) databases, and to open up further resources from other disciplines, as well in SocINDEX (sociology), GESIS (social sciences), and GReTA (gender studies). According to the terminological differences of the scientific disciplines and the different theoretical reference fields of the underlying four criteria, a wide range of keywords was used and combined in a variety of ways, e.g., “sex/gender”, “sex and gender”, “gender concept”, “sex/gender concept”, “gender framework”, “sex-linked biology”, “intersectionality”, “embodiment” (“ecosocial”, “biosocial”, “dynamic systems”), “gender equity”, “gender inequality”, “gender as process”, and “gender and relational theory”. The identification of relevant publications on theory-based concepts of sex/gender was continued until saturation was reached. The articles were sorted into the categories “conceptual sex/gender frameworks”, “case studies”, “guidelines for sex/gender based research”, and “operationalisations”.

Step 2: Evaluation of inclusion criteria. The sex/gender concepts available in the articles were reviewed by three researchers from gender studies to determine whether they reflected the four criteria multidimensionality, variety, embodiment and intersectionality in some or all respects. Only those articles based on at least one of the four criteria were further analyzed.

Step 3: Snowballing. In a snowballing process, the literature lists of the selected publications of step 2 were reviewed to identify additional relevant publications. In addition, literature from neuroscience sex/gender research which stands out through intensive methodological reflection combining standards from the natural sciences, the social sciences, and the humanities was added via the Neurogenderings Research Network (https://neurogenderings.wordpress.com/the-neurogenderings-network/, accessed on 21 October 2021).

After saturation had been reached through steps 1–3, a small number of compelling concepts (see Table 1) was selected based on expert judgement as a starting point for the development of our concept.

Step 4: Evaluation of consideration of sex/gender research requirements. The concepts the INGER researchers considered most crucial were summarized and qualitatively evaluated according to their contributions to the above-mentioned requirements of sex/gender research multidimensionality, variety, sex/gender interaction/embodiment, and intersectionality. Embodiment was divided into two sub-aspects, which are decisive for this approach. One is the concept of the physical and social dimensions of sex and gender as interacting inseparably, and the other is the insight into the consequences of this interaction, i.e., the biological incorporation of the social. An additional focus on power relations indicates whether the publication considers sex/gender only as an individual static characteristic or—in accordance with current gender research—as social processes at work at several levels of structural inequality and hierarchy [3]. Power relations are an overarching subject of analysis in gender research. Sex/gender should, therefore, not be regarded as a mere distinction of social groups, but as a term of analysis for positionings within a hierarchical social order. Criteria for evaluation include whether aspects of the requirements were only mentioned without in-depth explanation or whether comprehensive consideration or good elaboration was provided.

A total of 19 publications were identified which served as the foundational concepts and ideas for the operationalization of sex/gender that guided the development of the conceptual framework for quantitative environmental health research within INGER (Table 1). None of these 19 publications focused on environmental health. Ten publications provided theoretical concepts without any indication of possible operationalization [2,3,6,24,26,31,32,39,40,41], a further four publications included theoretical concepts and suggestions for operationalization [20,22,42,43]. Instructions for operationalization to differing degrees were given in another five publications [44,45,46,47,48]. With regard to the scientific discipline, thirteen publications were from health research [2,3,6,22,26,31,39,41,43,44,46,47,48], one from neuroscience [40], one from biology [24], two from social sciences [32,42], and two from psychology [20,45].

These previously published concepts or models of sex- and gender-related dimensions were evaluated according to the aforementioned four criteria, multidimensionality, variety, embodiment, and intersectionality, as shown in Table 1. Among the 14 publications on concepts or models, 4 considered all aspects which were assessed as relevant in recent biological and gender theory research [2,3,22,43], albeit at varying degree of detail and theoretical level. All in all, the publications provided important suggestions for a reformulation and meaningful dimensioning of sex and gender aspects in health research. Nine publications suggested approaches for operationalization of sex and/or gender to a varying degree [20,22,42,43,44,45,46,47,48]. The graphic model by Tate and colleagues [45] was a particular starting point for the development of the graphic elements in the INGER concept. In order to integrate a cisgender and transgender experiential spectrum of identity into studies, Tate et al. [45] proposed a “gender bundle” model which consists of five dimensions of gender, termed “facets”: (1) birth-assigned gender category (meaning sex assignment); (2) current gender identity; (3) gender roles and expectations; (4) gender social presentation; and (5) gender evaluations.

Two publications provided specific items to capture sex/gender in questionnaires [42,48]. Another two publications [46,47] referred to four well-established gender dimensions (gender roles, gender identity, gender relations, and institutionalized gender) originally made available in a primer of the Women’s Health Research Network in Canada [49] (adopted in an online tool of the Canadian Institutes of Health Research [50]; see also [22]) as a basis for constructing a gender index in secondary data analysis. Tannenbaum et al. [43] and Döring [42] cited examples of measures, referring to psychological scales, and Tannenbaum et al. [43] additionally pointed to the work of Smith and Koehoorn [47] and Pelletier et al. [46].

#### 2.2.3. Stage Three: Iterative Process of Concept Development and Refinement

In the third stage, we held a joint interdisciplinary discussion, during which we further evaluated those concepts for sex/gender, which were identified and selected during the second stage, with regard to their suitability for population health research and especially environmental health research. Based on these concepts and guided by gender theoretical considerations as well as further theories, such as Bourdieu’s habitus concept [51] and the doing gender approach [52], we developed a multidimensional sex/gender concept in an iterative process. The doing gender approach understands gender as a social process of interaction between people. Gendered identities, role realizations, and expressions of the self of an individual acquire meaning within the hierarchies and power relations of a social order. As such, gender develops its manifest reality by symbolic self-representation and the perception of others (e.g., names, clothing, voice pitch, gestures, facial expressions, behavior, posture, and body shapes). The habitus approach describes how people, depending on their membership in a social group, incorporate certain forms of practice, behavioral strategies, and norms, and thereby form a group-specific habitus, which can significantly shape health-relevant physical structures and processes. Identities, external expressions, and internalized roles reflect the social, cultural, and symbolic capital of individuals in a society (e.g., cultural skills, education, social relations, prestige, recognition) [51] and influence their social position in a structural and symbolic way.

This process of development of the sex/gender concept involved discussions and tentative operationalizations (1) within the interdisciplinary INGER research group; (2) together with the members of the scientific advisory board; and (3) together with the scientific community, when first reflections and drafts of the concept were discussed at workshops and conferences from 2018 to 2019 (see Appendix A). Comments and suggestions were incorporated into the sex/gender concept for further improvement. The iterative process took place from 2017 to 2020.

## 3. Results

INGER’s multidimensional sex/gender concept, with its intersectional perspective, was developed in an iterative process. It draws on manifold concepts, models, operationalizations and tools for sex/gender-related health research. Elements of the inventory of concepts or models of sex- and gender-related dimensions were modified or refined to meet the requirements of health research, especially environmental health research. The concept aims to fulfil the four established theoretical prerequisites to comprehensively study sex/gender aspects in population health research.

The multidimensionality and variety of sex/gender in its intersectional and embodied realization within the context of power relations is expressed in the graphic representation of the sex/gender concept. The sex/gender dimension includes three theoretical levels according to the gender theoretical standards described above: identity (individual self-concept), social structure (social position assignment, social sex-gender relations), and symbolic order (cultural meaning, cultural sex-gender relations). Arrows indicate influences, but not deterministic processes.

### 3.1. Multidimensionality

**Figure 1:**
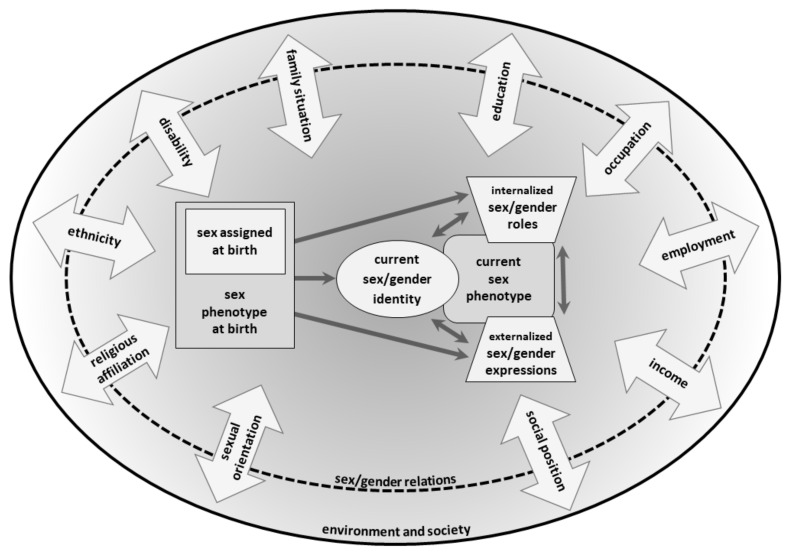
The INGER multidimensional sex/gender concept with its intersectional perspective.

In the center of the graphic representation of the concept (Figure 1), the individual sex/gender self-concept is illustrated with its five different dimensions: (1) sex phenotype at birth and sex assigned at birth; (2) current sex phenotype; (3) current sex/gender identity; (4) internalized sex/gender roles; and (5) externalized sex/gender expressions. At birth, the cultural process of sex assignment is carried out on the basis of the observable sex phenotype. This cultural interpretation of the body influences the further development of sex/gender without determining it (illustrated by the arrows with only one arrowhead). The developing sex/gender of an individual consists of further four distinctive dimensions: the current sex phenotype that consists of different biological components (e.g., gonads, hormones, genitals, and gender-related anatomy and physiology), the current sex/gender identity (e.g., non-binary, female/feminine), internalized sex/gender roles (e.g., own attitudes), and externalized sex/gender expressions (e.g., gendered behavior, appearance, clothing style, and other visual characteristics). The arrows with double arrowheads between the elements in the graphic representation depicting these four dimensions illustrate the interactions between them. The circle with the dashed line includes elements of the sex/gender self-concept at the individual level while showing that the sex/gender self-concept arises within the context of social and cultural sex/gender relations (e.g., social structures, such as the gendered division of labor or cultural norms shaping the notions of gender incongruence, masculinity, and femininity that influence all aspects of the individual self-concepts).

### 3.2. Variety

Sex phenotype, sex/gender identity, internalized sex/gender roles, and externalized sex/gender expressions are described as neither binary (male/masculine vs. female/feminine), nor as necessarily corresponding to each other (male phenotype is not always consistent with gender identification as a man, for instance). Instead, sex/gender is considered in its empirically reported variety. Therefore, the graphic representation leaves open which sex/gender constellations as a combination of the several elements could occur. That includes the differentiation of sex phenotypes (for example, variety within sexed groups regarding hormonal states, gonadal traits, or metabolism, as well as intersex conditions), gender identity (such as trans, male/masculine, non-binary, female/feminine), gender roles, and gender expressions (for instance, masculinized behavior with feminine expression). Furthermore, the different dimensions of sex/gender can be joined in various ways (e.g., female phenotype in combination with masculine gender roles and expressions).

### 3.3. Embodiment

In order to show interdependency and causal entanglement of the social and physical dimensions of sex/gender, the graphic boxes which symbolize current physical, social, and cultural characteristics are drawn as mutually overlapping fields. The sex phenotype can be understood as a possible way of bodily expression within a genetically predetermined reaction norm that takes on different realizations depending on social behavior and context (testosterone level in men and women fluctuates depending on physical activity and parental care, for instance). Gender dimensions, in turn, may also be influenced by physiological processes and bodily expressions (e.g., muscle strength, that is itself influenced by gender norms and practices such as gendered training preferences, can, in turn, influence gendered behavior, for example physical dominance).

### 3.4. Intersectionality

Sex/gender is conceptualized as interacting with other categories of social inequality and power relations (such as experience of gendered discrimination due to socioeconomic position or ethnicity). The wide arrows with double arrowheads in the graphic representation list some of these intersecting social categories or power relations without any claim to completeness. The arrows crossing the circle with a dashed line point to the fact that structural sex/gender relations as well as the individual sex/gender self-concepts are shaped by further social categories and power relations. Thus, these arrows represent different social identities and positions that are linked with privileges or disadvantages.

## 4. Discussion

Based on gender theoretical and health science approaches, we developed a multidimensional sex/gender concept with an intersectional perspective as a basis for sex/gender operationalization in data collection and analyses in quantitative health research, especially environmental health research. This concept considers the four criteria—multidimensionality, variety, embodiment, and intersectionality—in order to comprehensively study sex/gender aspects in population health research. The development of our concept was inspired by previously published concepts or models of sex- and gender-related dimensions.

### 4.1. Relevant Previous Sex/Gender-Concepts

Four previous seminal publications [2,3,22,43] met those four quality criteria to a great extent. The four criteria, i.e., multidimensionality, variety, embodiment, and intersectionality, were met by the definitions of sex and gender [2,22,43] or by suggestions for operationalizations, for instance considering inter* and trans* persons. Despite achieving all criteria and making important contributions to sex/gender research in the health sciences, in our view, some aspects were not elaborated in sufficient detail to guide researchers towards a comprehensive integration of sex/gender in quantitative theory-based health research. In addition, none of these publications presented a graphic model.

In her tutorial on “genders, sexes, and health”, Krieger considers essential aspects of the concept intersectionality when she refers to “social inequalities among women” [2] (p. 653) and “social divisions premised on power and authority (e.g., class, race/ethnicity, nationality, religion)” [2] (p. 653), but she does not explicitly elaborate the concept of intersectionality. In their report on a primer aimed to improve understanding and usage of the concepts of sex and gender in health research, Johnson et al. [22] include sex and gender as respective continua in their definition, though their examples of different sex/gender dimensions which apply only the binary positions of “male” and “female” and “men” and “women”. The only exception is the dimension of gender identity where inter* and trans* identities are taken up. The term embodiment by itself was not mentioned. The authors conceptualize sex and gender analogously as distinct but “inextricably linked” [22] (p. 4) (for instance, gender affects sex: high-risk activities increase testosterone levels). However, the concept’s underlying incorporation of the social world and social inequalities is not treated. Tannenbaum et al. [43] (p. 1) describe the rationale for and the “application of methods for integrating sex and gender in implementation research”. Though Tannenbaum et al. [43] include the criterion variety of sex and gender through trans* and inter* persons at the level of operationalization, the dimensions of sex and gender are only defined in binary terms by referring to “males” and “females” or “men” and “women”. Sex and gender are considered as “interrelated, interactive, and potentially inseparable” [43] (p. 3) but no conceptualization of the entanglement, such as embodiment, is provided. Intersectionality is only addressed in terms of “intersecting variables” without further elaboration of the underlying concept [43].

A comprehensive theoretical grounding of the four underlying criteria is given by Hammarström et al. [3] in their presentation of the state of the art of central gender theoretical concepts in health research. After a short introduction to the gender theoretical concepts of sex, gender, intersectionality, embodiment, as well as gender equality and gender equity, the authors present a discussion of an appropriate, theory-based use of these concepts in health research in which all criteria are considered. The INGER conceptual framework explicitly takes up four of these concepts (sex, gender, intersectionality, embodiment) and is compatible with concepts of gender equality and gender equity when referring to discrimination with regard to sex/gender and to the broader context of health equity, respectively. To us, only the symbolic level seemed to not be comprehensive enough, so that, in addition, the INGER multidimensional sex/gender concept considers a symbolic level in accordance with the habitus approach [51] from sociology to conceptualize individual identities, preferences, and behaviors within the framework of a social structure.

### 4.2. Theoretical Foundation of INGER’s Multidimensional Sex/Gender-Concept

Each sex/gender dimension of the INGER concept proves to be compatible with the model of the Women’s Health Research Network in Canada, as adopted by the Canadian Institute of Health Research, which comprises gender roles, gender identity, gender relations, and institutionalized gender [22], so that sex/gender can be analyzed as an individual and structural category. However, INGER differs to previous applications in that, according to the doing gender approach [52] and the habitus approach [51], the dimensions of sex and gender at the individual level are always placed into a context of social and symbolic structures that shape them. This embedding of the gendered individual in structural and symbolic contexts counteracts the tendency towards holding individuals responsible for their health and ignoring the underlying structural conditions of individual action.

Since gender can be shaped differently depending on societal context, norms, and cultural values, accordingly, sex/gender identities, roles, and modes of representation can differ not only between but also within sex/gender groups. In this way, the habitus concept is directly linked to the intersectionality approach. Within the INGER conceptual framework, intersectionality is integrated with respect to the social science multi-level approach of Winker and Degele [32]. This approach implies that the object of research is not primarily a multi-discriminated individual, but rather the functioning of the social structure in which an individual is socialized. In recent years, publications within health sciences have already addressed the consequences of structural social inequalities and power relations for a quantitative intersectionality approach [31,53,54,55,56]. Quantitative analyses of intersectionality are often based on McCall’s inter- and intra-categorical approach [57], defining categories of difference (e.g., sex/gender, race/ethnicity, class, sexuality), and analyzing which differences exist within (intra-categorical) and between (inter-categorical) groups with regard to health outcomes. The challenge, however, is to conceptualize differences not only as group-specific attributions on an individual level, but to identify the underlying causes of these differences at a contextual and structural level. Recent reviews have emphasized the limitations of studies with a quantitative intersectionality-based approach when interactions between categories of difference were statistically analyzed only in a simplistic way on an individual level without reasonable theoretical foundation and without taking structural aspects and mechanisms of privilege and disadvantage into account [55,56].

### 4.3. The INGER Sex/Gender-Concept in Relation to Previous Graphic Sex/Gender Concepts

For the development of our INGER conceptual framework, we also evaluated previous publications providing graphic representations of sex/gender models. As mentioned in the Methods Section, the INGER model was, in part, inspired by the gender bundle proposed by Tate and colleagues [45], which divides the concept of gender into five different facets. INGER adopted and modified some of these facets. One limitation of the gender bundle is that it does not comprise physiological sex/gender aspects and does not integrate the concepts of embodiment and intersectionality.

Schellenberg and Kaiser [20] aimed to provide a basis for discussion of possible strategies for nonbinary sex/gender conceptualizations in psychological studies. They depicted a model of sex/gender as a multidimensional concept with six dimensions. They call their approach of six sex/gender dimensions assessed by questionnaire in conjunction with steroid assessment as a hormonal component “sex/gender battery”. The graphical representation of the sex/gender dimensions comprises (1) “sex/gender traits”; (2) “sex/gender role behavior”; (3) “sex/gender attitudes”; (4) “sex/gender identification”; (5) “sex/gender expression”; and (6) “(recalled) sex/gender socialization” [21] (p. 180). Except for dimension number four, these dimensions relate to the structural and symbolic level. The authors refer to the approach of sex/gender mosaicism [40]. According to this approach, individuals are not exclusively feminine or masculine in traits, roles, and attitudes. Furthermore, it is not possible for an individual to have a “single-sex” phenotype. In this way, they avoid the reduction of sex/gender to a binary category. Although they refer to multidimensionality and variability of sex/gender as well as to aspects of the embodiment concept and to intersectionality in the theoretical background, the last two aspects are not further expanded on and are not included in their model graph.

What is often missing in (graphic) sex/gender concepts is a consideration of the physical dimension of sex/gender based on recent findings in developmental biology. Due to our research focus on environmental health, we integrated this dimension in our sex/gender concept as “sex phenotype”. This phenotype can be seen as plastic ensemble of traits within the range of a genetically determined reaction norm, which allows different specifications depending on the environment (following the biologists [23,24,58]). On this basis, sex is no longer understood in a simplistic way as an essentialist expression of physically determined gender. Therefore, health situations related to sex/gender can be analyzed on the level of physiological processes in a social context. Likewise, gender is no longer seen as a body-indifferent, purely social dimension detached from biological processes, which means that gender identities, gender roles and norms, and the realization of social gender relations in everyday practices are always thought of in connection with their health-relevant embodiments.

### 4.4. Recent Developments of Sex/Gender Conceptualisation in Health Sciences

The conceptual framework of INGER aims to contribute to the recent increase in awareness in health sciences that sex/gender is a non-binary and complex social determinant of health that varies according to time and place [1,5,59]. During the same period that INGER’s conceptual framework was developed, Heise and colleagues [5] created the “conceptual framework of the gender system and health”. It is based on similar theoretical foundations and largely implements similar elements in its graphic representation, albeit the conceptual frameworks follow different purposes. While the INGER conceptual framework aims to make key causal effects of socialization applicable to operationalization in quantitative studies on health, the model by Heise et al. [5] provides a fundamental overview of the mechanisms and gendered pathways that lead to health inequities. Common to both conceptual frameworks is the consideration of the life course perspective: a person is born with a biologically pre-structured body in the context of gender-specific power relations and norming instances that are assigned to specific social positions through intersection with other axes of power and privilege. Both conceptual frameworks also include a lifelong context sensitivity of the body and emphasize the inseparability of the physical and the social (of sex/gender).

Slight differences between the two conceptual frameworks exist in the reference to the social dimensions of gender. While Heise et al. [5] depict the mechanism of socialization itself by symbolizing its structural and symbolic influencing factors as interlocking gears, the INGER conceptual framework integrates central causal effects of socialization in the graphic boxes in order to provide starting points for operationalization in quantitative studies on health.

Another difference concerns the way that the models deal with sex/gender diversity and intersectionality. While Heise et al. [5] present males and females with intersectional internal differentiations of these groups, there is no reference to sex/gender binary in the INGER model. From an intersectionality perspective, a multitude of categories of social inequalities and power relations are shown in the model without any claim to completeness. In contrast, Heise et al. [5] argue that race, class, age, and ability define gendered social positions. The overarching bar with structural and social determinants of health might be interpreted in terms of further intersectional categories.

Further recent scientific developments relate to gender indices or gender-related variables. For example, Ballering et al. [60] created a gender index retrospectively based on the pre-existing data of a cohort study. The particular initial situation may be described by availability of data characterizing biological aspects of the sex dimension as well as of gender-related data covering gender roles, gender relations, and institutionalized gender. Interestingly, those participants with an intersex condition (defined as chromosomal variation of the sex chromosomes, genetic mutations leading to hormonal disturbances, or deviations of genital organs), and those participants with non-conforming gender identity (e.g., using prescribed hormones) were excluded from the analysis to construct the gender index. The authors themselves state that they included “the most stereotypic women and men from a biological viewpoint” [60] (p. 2). The gender index included the most discriminative gender-related variables regarding the dichotomous sex variable. Thus, the composite gender index classified individuals on a bipolar continuum ranging from of masculine to feminine. The authors follow a different approach than INGER’s comprehensive analysis of sex/gender inequalities in health based on multidimensionality, variety, embodiment, and intersectionality.

Another recently published example is the approach of Nielsen et al. [61]. The authors distinguish between sex as a biological variable and gender as a sociocultural variable (while acknowledging interactive effects of sex and gender on health) and developed a gender assessment tool, termed “Stanford Gender-Related Variables for Health Research”. Seven gender-related variables were identified: caregiver strain, work strain, independence, risk-taking, emotional intelligence, social support, and discrimination [61]. These aspects might be interpreted as solution-linked variables, indicating modifiable societal and contextual factors from an intersectionality perspective [56,62]. However, Nielsen et al. [61] conceptualized these gender-related variables primarily as indicators of individual behaviors and attitudes attributed to a gender group, thereby laying less focus on social power relations and societal context. Because this tool presumably disregards the entanglement of sex/gender, embodiment, the structural dimension of gender, and the life-course perspective, it does not add further aspects to the INGER conceptual framework.

### 4.5. A Call for Theory-Based Health Inequalities Research

There has been a call for a deeper engagement with theory to develop well-defined concepts and more complex, dynamic models to improve understanding of the mechanisms and pathways linking social structures and processes to health inequalities [3,63,64,65,66]. The collaborative research project INGER aimed to make mutual use of theory from different scientific disciplines to create a multidimensional sex/gender concept with an intersectional perspective. This conceptual framework was developed to be the basis for the operationalization of sex/gender in quantitative health research, especially for research questions on environmental health. Due to the ambitious and comprehensive model, several challenges arose.

To indicate a few: Obviously, an exhaustive list of the dimensions and components, which should be considered when measuring sex/gender beyond the binaries cannot be made available on a general level in advance. The specific research questions of a study should guide the choice of relevant items against the background of gender theoretical foundations. For the assessment of internalized sex/gender roles and externalized sex/gender expressions, it is essential to avoid stereotypes in data collection while assessing self-perception as well as external perception in a given context of social norms and cultural values. Societal changes and dynamics might further contribute to this challenge. Assessing the process of embodiment of socioeconomic and psychosocial conditions constitutes another challenge. Krieger [67] (p. 222) emphasizes that embodiment is an active and reciprocal process of biologically incorporating the outer world, being “more than just about how social conditions “get under the skin” ”. Within ecosocial theory, embodiment “emphasizes the multilevel and dynamic interplay between processes and structures relevant for health and the production of population health inequalities” [3] (p. 188). The conceptualization of constrained choice by Bird and Rieker [68], as well as innovative approaches to measure biological mechanisms, such as allostatic load [29], might contribute to the assessment. Moreover, Krieger [28] (p. 41) provides an overview of the “types of measures of unjust isms, and anti-isms, for health equity research and implications for study design”. She distinguishes as exposure levels the structural, individual, and internalized level and discusses selected examples of measures so far in health equity research. According to Krieger [28], pathways of embodiment relevant to exposure to “unjust isms” comprise amongst others social and economic deprivation, social trauma in the form of discrimination, or exposure to environmental hazards. Finally, from an intersectionality perspective, sex/gender has to be analyzed in relation to other categories of social identities and positions, power structures at the contextual level, societal systems of domination and privilege, and processes of discrimination [3,28,65]. Intersectionality contributes to the identification of the ways that multiple dimensions of social inequalities simultaneously influence population health (inequities). “An intersectional lens shows that while health is experienced at the level of the individual, individual health outcomes and inequities, manifested in the body, are inextricably linked to interacting processes and structures of power at multiple levels.” [26] (p. 11). Recent reviews gave an overview of quantitative research applications of intersectionality, which could help researchers improve future research [55,56].

### 4.6. Upcoming Research Activities in INGER

With regard to INGER, the implementation of the multidimensional sex/gender concept with its intersectional perspective has recently been tested in two quantitative studies on environmental health. In the next step, comprehensive data analyses will be performed to show how this conceptual framework might help to gain new insights about significance of sex/gender in environmental health and, thus, could have an impact on practice and policymaking of environmental health promotion and protection.

## 5. Conclusions

In conclusion, the conceptual framework developed by the interdisciplinary team of the collaborative research project INGER is intended to provide a theoretically sound starting point for the operationalization of sex/gender in quantitative health research. The conceptual framework aims to provide help with the complexity and dynamics of sex/gender, thereby preventing an inadequate consideration of sex/gender in health sciences based on everyday ideas about social and biological dimensions, gender stereotypes, and assumptions of a binary static category. By building upon the latest state of research of several disciplines, the conceptual framework will significantly contribute to the integration of gender theoretical concepts into (environmental) health research, thus improving the validity of research and supporting the promotion of health equity in the long term.

## Figures and Tables

**Table 1:** Concepts or models of sex- and gender-related dimensions for quantitative research.

Focus of Content *	Authors/Year (Ascending)	Basic Approach	Multidimensionality		Variety		Embodiment		Intersectionality	Power Relations
			Gender	Sex	Gender	Sex	Sex/Gender Interaction	Incorporation of the Social		
T	Krieger (2003) [2]	Framework for the study of the connection between gender, sex and health	++	++	++	++	++	++	+	++
T	Nieuwenhoven and Klinge (2010) [39]	Introduction to sex- and gender-sensitive research and step-by-step plan to consider sex and gender in all research phases	+	++			+	+		+
T	Winker and Degele (2011 [32])	Theory and method of intersectional, qualitative multi-level analysis	++	+	++	+			++	++
T	Fausto-Sterling (2012) [24]	Introduction to sex and gender from a developmental biological perspective placed in an historical and cultural framework	++	++	++	++	++	++		+
T	Springer et al. (2012) [6]	Theoretical frame and good practice guidelines for researching sex/gender in human health	++	+		+	++	+	+	
T	Bauer (2014) [31]	Discussion of potential and challenges of incorporating intersectionality theory into population health research methodology					++	++	++	++
T	Hammarström et al. (2014) [3]	Comparison and discussion of six gender theoretical concepts in health research	++	++	++	++	++	++	++	++
T	Rippon et al. (2014) [40]	Implications of four key principles for research process derived from sex/gender conceptualization; guideline for sex/gender research in neuosciences	++	++			++	++	+	++
T	Schiebinger and Klinge (2015) [41]	Seven methods suggested for sex and gender analysis	++	++	+	+	+	+	+	
T	Hankivsky et al. (2017) [26]	Combining biological approaches and intersectionality			+	+	+	++	++	++
T/O	Johnson et al. (2009) [22]	Review of practical suggestions for the application of sex and gender in health research	++	++	+	++	++	+	++	++
T/O	Döring (2013) [42]	Operationalisation of sex/gender	+	++	++	++			+	+
T/O	Tannenbaum et al. (2016) [43]	Integration and measurement of sex and gender in implementation research	++	++	+	+	+	+	+	++
T/O	Schellenberg and Kaiser (2018) [20]	Strategies for multidimensional and non-binary sex/gender conceptualisations and measurements	++	++	++	++	++		+	++
O	Phillips (2008) [44]	Development of a proxy measure for gender (indicator of gender role acceptance and effects of gender inequity)	++					+	+	++
O	Tate et al. (2014) [45]	Differentiation of gender into five components	+		++		+			
O	Pelletier et al. (2015) [46]	Differentiation of sex and gender and data-based creation of a gender index	++					+		++
O	Smith and Koehoorn (2016) [47]	Data-based construction of a gender index	++					+		+
O	Bauer et al. (2017) [48]	Trans inclusive measurements of sex/gender	+		++	+			++	

* (T) Theoretical Concepts/models, (T/O) Theoretical concepts with operationalization, (O) Operationalization. Legend of evaluations: ++ comprehensive consideration or good elaboration of the aspect, + Mention of the aspect without in-depth explanation.

## Data Availability

Not applicable.

## Appendix A

Attended workshops and conferences to discuss first drafts of the INGER multidimensional sex/gender concept.

**Workshops**
2018: Gender diversity in survey research, University of Gothenburg, Sweden
2019: Classification in the era of personalized medicine: perspectives from history, philosophy and policy, Humboldt-University Berlin, Germany
2019: Inequalities in environment-related health protection and health promotion, University of Bremen, Germany

**Scientific conferences**
2018: National conference of the German Society for Epidemiology, Bremen, Germany
2019: International conference of the International Society for Environmental Epidemiology, Utrecht, The Netherlands
